# Editorial: Cryptogenic ischemic stroke

**DOI:** 10.3389/fneur.2025.1692475

**Published:** 2025-09-08

**Authors:** Antonio Arjona-Padillo, Laura Amaya-Pascasio, Elena Meseguer, Patricia Martínez Sánchez

**Affiliations:** ^1^Department of Neurology, Torrecárdenas University Hospital, Almería, Spain; ^2^Assistance Publique Hôpitaux de Paris, Paris, France; ^3^Health Research Center (CEINSA), Faculty of Health Science, University of Almería, Almería, Spain

**Keywords:** biomarkers, cryptogenic stroke, ESUS, thrombophilia, ischemic stroke, foramen ovale

Cryptogenic ischemic stroke, an imaging-confirmed ischemic event with no identifiable etiology despite a comprehensive workup (1), and its subtype, embolic stroke of undetermined source (ESUS) (2), remains a challenging diagnosis for vascular neurologists. The Research Topic featured in *Frontiers in Neurology* includes several articles about cryptogenic stroke, they describe uncommon etiologies and guide the selection of optimal therapeutic strategies in the absence of a definitive cause.

Potential causes of cryptogenic stroke can be classified according to the system involved. Cardioembolic sources include left atrial pathology (e.g., covert atrial fibrillation, atrial cardiomyopathy), left ventricular disease, patent foramen ovale (PFO), and valvular disease (3). In this context Zhang et al. describes the use of dual stent retrievers in mechanical thrombectomy for infective endocarditis-associated large vessel occlusion, a strategy that is particularly useful in cases of bifurcation occlusions.

In this Research Topic Saito et al. present findings of a prospective multicenter substudy in Japan, they included 241 patients with recent ischemic stroke, and evaluated a non-invasive, wireless patch ECG during seven days to detect atrial fibrillation (AF). This arrhythmia was detected in 8.7% of patients. Although this rate is lower than in other cohorts (4), the simplicity and accessibility of this diagnostic method are noteworthy.

Three contributions address the relationship between PFO and stroke. Two narrative reviews delve into pathophysiological mechanisms such as *in-situ* thrombosis, paradoxical embolism, atrial cardiopathy, arrhythmias, circulating biomarkers, and genetic predisposition (Li et al.; Amini). Shah et al., proposes a novel diagnostic approach employing transcranial Doppler (TCD) supported by an AI-assisted robotic system. Using transesophageal echocardiography (TEE) as the reference standard for PFO detection, TCD achieved 92% sensitivity and 87.5% specificity in detecting large right-to-left shunts; by contrast, TTE yielded 78.6% and 71.4%, respectively. Discordance between TCD and echocardiographic findings is not uncommon, potentially due to differences in shunt origin or Valsalva maneuver efficacy during TEE (5). These results support the use of TCD a screening tool for PFO detection.

Esnaola Barriola et al. report a case series of 10 patients with mobile aortic arch thrombi associated with atherosclerotic plaques, identified via suprasternal echocardiography performed by neurologists. The patients received anticoagulation, although optimal management strategies remain unclear (6). Follow-up imaging was also included in this study.

Non-stenotic carotid atherosclerosis is an underrecognized etiology of ischemic stroke, warranting a multimodal diagnostic approach. High-resolution MRI can assess plaque vulnerability and therapeutic response, as demonstrated in a case series by Yang et al.. Additionally, Zhou and Hui conducted a systematic review on contrast-enhanced ultrasound (CEUS) for detecting unstable plaques. Although CEUS demonstrated moderate sensitivity and limited specificity (61%), it remains a cost-effective and accessible alternative compared to advanced imaging modalities.

Acquired hypercoagulability, especially cancer, represents another relevant category in cryptogenic strokes. Nearly 15% of oncology patients experience stroke, and 2–10% are diagnosed with stroke annually. Carneado-Ruiz's narrative review emphasizes diagnostic biomarkers, particularly D-dimer, and treatment considerations in oncologic patients with ischemic stroke. Although anticoagulation is often selected, its superiority over antiplatelet therapy remains unproven, except in specific contexts such as venous thromboembolism with PFO, nonbacterial thrombotic endocarditis, and markedly elevated D-dimer (7). Moreover anticoagulants, including direct oral agents, may increase bleeding risk in this population (8). Diagnostic strategies for cancer-related stroke, including optimal timing and modality, are yet to be standardized.

Biomarker research continues to expand. Beyond D-dimer, cardiac biomarkers such as B-type natriuretic peptide (BNP) and N-terminal pro-BNP (NT-proBNP) are proposed as markers for ESUS of cardioembolic origin (9). Hang et al. applied Mendelian randomization and genome-wide association studies (GWAS) to assess causal relationships between metabolites and ESUS. Their findings suggest a protective association with O-methylascorbate (X-11593) and implicate various lipid-related metabolites. However, generalizability is limited to European populations, and further research is warranted.

Finally, Ruiz-Franco et al. examined neurological manifestations in a cohort of Fabry disease patients, assessing the prevalence of cryptogenic stroke as a potential early manifestation (10). No association was found, likely due to regional genetic differences or enzyme replacement therapy effects.

In summary, the contributions in this Research Topic provide valuable insights into the multifactorial nature of cryptogenic ischemic stroke and underscore the need for continued multidisciplinary research to refine diagnostic and therapeutic strategies.
